# Posterior fossa arachnoid cysts in multiple system atrophy

**DOI:** 10.1016/j.ibneur.2025.11.015

**Published:** 2025-11-28

**Authors:** Audrey M. Blazek Ramsay, Eoin P. Flanagan, Elizabeth A. Coon, Lauren M. Jackson

**Affiliations:** Department of Neurology, Mayo Clinic, Rochester, MN, USA

**Keywords:** Multiple system atrophy, Arachnoid cyst, Space-occupying lesion, Neurodegenerative disease

## Abstract

**Background:**

Multiple system atrophy (MSA) is an α-synucleinopathy characterized by prominent cerebellar or parkinsonian features. This is pathologically due to the deposition of α-synuclein in glial cells, the mechanism by which s not entirely clear. We describe three patients with posterior fossa arachnoid cysts later diagnosed with clinically established MSA, suggesting the co-existence of two rare pathologies through a shared mechanism versus by chance.

**Cases:**

Patients ranged from 45 to 67 years of age at presentation, each describing 2–3 years of progressive symptoms. All displayed cerebellar findings and various non-motor features of orthostatic hypotension, genitourinary failure, and dream enactment behavior confirmed on diagnostic testing or on a clinical basis. All imaging demonstrated posterior fossa arachnoid cysts and supportive features of MSA, notably pontine and cerebellar atrophy.

**Conclusions:**

The probability of having both MSA and a posterior fossa arachnoid cyst based on population study-derived point prevalence and assuming independence of the events is 1.09 e-6. We suspect that while uncommon, concurrence of the diseases is due to chance. It is plausible that space-occupying lesions, when already present, may contribute to neurodegenerative disease progression.

## Introduction

The diagnosis of multiple system atrophy (MSA) requires core features of autonomic dysfunction and a predominant parkinsonian or cerebellar syndrome, as defined by the 2022 Movement Disorder Society (MDS) criteria (Wenning et al., 2022). Individuals with the parkinsonian subtype, or MSA-P, may exhibit bradykinesia, rigidity, or tremor, while those with the cerebellar subtype, or MSA-C, may exhibit gait or limb ataxia, cerebellar dysarthria, and oculomotor dysfunction (Wenning et al., 2022). Supporting MRI findings include atrophy or increased diffusion signal of the middle cerebellar peduncle, putamen, pons, and cerebellum (Wenning et al., 2022, Monzio Compagnoni and Di Fonzo, 2019). Symptoms typically arise in the 6th decade and often rapidly progress within 3 years (Monzio Compagnoni and Di Fonzo, 2019). Prognosis is poor, with mean survival under 10 years from symptom onset in both MSA-P and MSA-C (Monzio Compagnoni and Di Fonzo, 2019, Low et al., 2015).

Pathologically, MSA is associated with α-synuclein (α-syn) aggregation with neuronal loss (Monzio Compagnoni and Di Fonzo, 2019, Low et al., 2015). Several hypotheses regarding the abnormal intracellular collections of α-syn in MSA have been raised: α-syn overexpression, uptake of α-syn by glial cells, α-syn accumulation in response to glial-produced proteins, decreased or impaired α-syn degradation, “prion-like” spread of α-syn with various strains of different aggressiveness, and mitochondrial dysfunction and resultant inflammation (Monzio Compagnoni and Di Fonzo, 2019). Multiple mechanisms may contribute to the degree of neuronal loss and clinical phenotype.

Arachnoid cysts are incidental findings found in 1.4 % of the population (Al-Holou et al., 2013). There is limited evidence whether posterior fossa cysts with mass effect may be associated with the development of a neurodegenerative disease (Bachstetter et al., 2021).

We describe three patients with posterior fossa arachnoid cysts and clinically established MSA. We calculate the probability of having both a posterior fossa arachnoid cyst and MSA by chance and describe possible relationships between the two pathologies.

## Case series

**Fig. 1:**
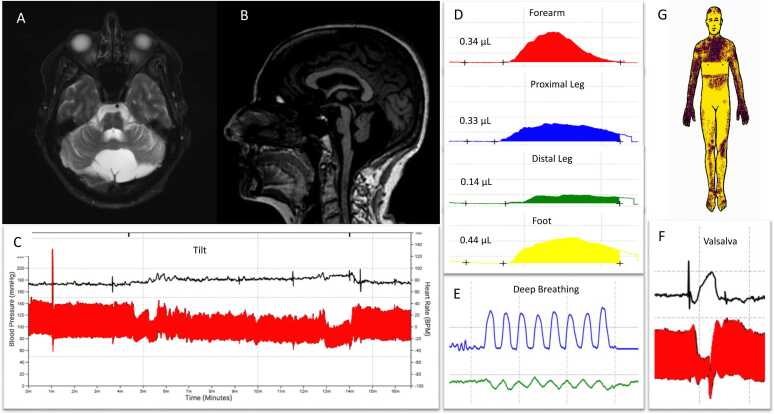
Axial T2 MRI demonstrates a retrocerebellar cyst and hot cross bun sign (A). Sagittal T1 shows advanced olivopontocerebellar atrophy (B). Tilt (C), postganglionic sweating (D), and deep breathing (E) and Valsalva (F) responses are normal. TST demonstrates 63 % anhidrosis (G, yellow).

Case 1. A 67-year-old woman with multiple sclerosis (MS), deemed inactive, presented with 3 years of progressive ataxia, dysarthria, and dream enactment behavior. Other symptoms included emotional lability (sudden crying), new urinary urge incontinence otherwise not explained by MS, and constipation. Surveillance imaging over 6 years revealed a stable 8.3 × 2.9 cm posterior fossa arachnoid cyst and severe, progressive olivopontocerebellar atrophy with a “hot cross bun” sign (Fig. 1). There was no evidence of new inflammatory lesions attributed to MS on imaging. Examination showed ataxic dysarthria, nystagmus, mild parkinsonism, with severe truncal and moderate appendicular ataxia. Thermoregulatory sweat test (TST) was abnormal with widespread anhidrosis (63.8 %) in a central pattern. Autonomic reflex screen was normal, though given unexplained urinary urge incontinence she met criteria for clinically established MSA-C.

**Fig. 2:**
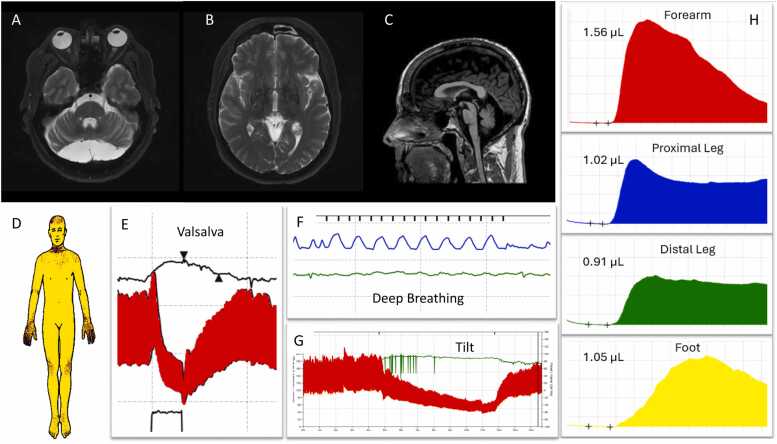
Axial T2 MRI demonstrates a retrocerebellar cyst (A) and putaminal rim sign (B). Sagittal T1 shows pontine and cerebellar peduncle atrophy (C). TST shows global anhidrosis (94.5 %) in a central pattern (D). There is severe cardiovascular adrenergic impairment with Valsalva (E) and deep breathing (F). Tilt shows neurogenic orthostatic hypotension (G). Postganglionic sweating is normal (H).

Case 2. A 53-year-old man with autosomal dominant myotonia congenita presented with 2 years of progressive imbalance and orthostatic hypotension with syncope. Other symptoms included several years of urinary incontinence and recurrent urinary tract infections, constipation, erectile dysfunction, dream enactment behavior, dysphagia, and pseudobulbar affect. Examination showed hypophonic/spastic dysarthria, moderate parkinsonism, and moderate appendicular and gait ataxia. Imaging revealed pontine and cerebellar peduncle atrophy with a putaminal rim sign, and large posterior fossa arachnoid cyst (Fig. 2). TST showed global anhidrosis (94.5 %) in a central pattern, with acral and forehead preservation of sweating. Autonomic reflex screen showed severe cardiovascular adrenergic impairment and neurogenic orthostatic hypotension. He was diagnosed with clinically established MSA and unfortunately died at the age of 54.

**Fig. 3:**
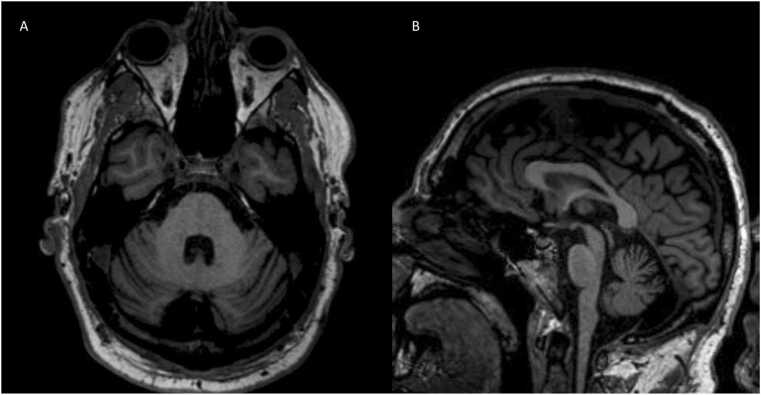
Axial gradient echo demonstrates a retrocerebellar cyst and cerebellar atrophy (A). Sagittal T1 shows pontine and cerebellar atrophy (B).

Case 3. A 45-year-old man presented with 2 years of progressive parkinsonism, urinary retention, and incontinence. Other symptoms included orthostatic hypotension, erectile dysfunction, and stridor. Examination showed hypophonic/spastic dysarthria, moderate parkinsonism, and moderate axial and appendicular ataxia. Imaging showed moderate cerebellar atrophy and a small retrocerebellar arachnoid cyst (Fig. 3). Post-void residual was 400 cc. A skin biopsy was positive for α-syn at all sites. Autonomic testing at an outside hospital showed neurogenic orthostatic hypotension. He was diagnosed with clinically established MSA and unfortunately died at the age of 45.

## Discussion

We present three patients with posterior fossa arachnoid cysts who were diagnosed with MSA, all with clinical cerebellar involvement.

Derived from a population study, the point prevalence rate for MSA at age ≥ 40 years is 7.8:100,000 and for posterior fossa arachnoid cysts is 1400:100,000 (Al-Holou et al., 2013, Bjornsdottir et al., 2013). Assuming the two events are independent, the probability of an individual having both a posterior fossa arachnoid cyst and MSA by chance is 1.09 e-6. Using a United States population of 160.7 million for age ≥ 40 years, we expect 175 cases of co-existent posterior fossa arachnoid cyst and MSA (Bureau, 2023).

This calculation shows that on a population level, concurrent posterior fossa arachnoid cyst and MSA may be a chance event. A similar study evaluated the probability of a husband and wife both developing MSA, and despite an estimated 0.25 conjugal cases of MSA in the US and 23 cases in the world, chance was the best explanation for their co-existence with possible contribution from shared exposures (Coon et al., 2019). While not indicative of causation, our case series raises the question of whether space-occupying lesions can contribute to the development or progression of neurodegenerative disorders. We propose that based on the probability calculation, it is more convincing that the two disorders occur together by chance. However, the presence of a cyst contributing to disease progression is plausible.

The development of tau astrocytopathy in the setting of long-standing arachnoid cysts has been reported (Bachstetter et al., 2021). It is proposed that chronic mechanical stress, as in repetitive trauma or space-occupying lesions, may result in degenerative changes of surrounding astrocytes in patterns different from typical neurodegenerative diseases but manifesting in similar phenotypes. In alpha-synucleinopathies, inflammation and oxidative stress, as can be seen in chronic traumatic brain injury, has been shown to lead to the development of parkinsonian symptoms due to alpha-synuclein overexpression (Acosta et al., 2015). Chronic traumatic encephalopathy is also associated with various types of neurotrauma and is characterized by perivascular deposition of phosphorylated tau in neurofibrillary tangles deep in cortical sulci, followed by spread of abnormal protein (McKee et al., 2015). Other proteins that are known to accumulate include 43 kDa TAR DNA-binding protein and amyloid beta. This protein deposition can lead to prominent behavioral change among other symptoms representing neurodegenerative disease depending on the location of accumulation and degree of spread. While the exact mechanism of protein deposition in unknown, it is proposed that repetitive stretch forces may damage blood vessels and axons in the area of trauma. Despite differences in the specific protein - α-syn in MSA – the mechanisms of protein aggregation and spread may be similar in patients with space-occupying lesions, with chronic alteration in local brain architecture.

Ninety-seven percent of retrocerebellar arachnoid cysts are asymptomatic due to the ability of surrounding tissues to adjust and compensate for slow growth (Bachstetter et al., 2021). Symptoms of cranial neuropathies, headache, appendicular and gait ataxia, and cognitive and psychiatric changes have been described in adult and pediatric populations (Al-Holou et al., 2013). The degree of cerebellar symptoms and rate of progression in the three patients presented is felt to be due to MSA and out of proportion to what is expected from a posterior fossa arachnoid cyst alone. In a series of only 3 patients, we cannot make conclusions on the rate of progression of disease. It is worth noting that the patient in Case 3 died within 2 years of disease onset, more rapid than the median survival from symptom onset to death (Monzio Compagnoni and Di Fonzo, 2019, Low et al., 2015).

The co-existence of two rare disorders suggests there may be a shared mechanism in their pathophysiology. While the most likely explanation for co-occurrence is chance, increased susceptibility to α-syn accumulation and neuronal loss due to cerebellar and brainstem compression in the setting of an arachnoid cyst is an interesting concept. Further research can be done to better elucidate the relationship between space-occupying lesions and the development and progression of neurodegenerative disease.

## Ethical compliance statement

This study was exempt from the Mayo Clinic Institutional Review Board. Informed patient consent was not necessary for this work. We confirm that we have read the journal’s position on issues involved in ethical publication and affirm that this work is consistent with those guidelines.

## Funding sources

This research did not receive any specific grant from funding agencies in the public, commercial, or not-for-profit sectors.

## Financial disclosures for the previous 12 months

The authors declare that there are no additional disclosures to report.

## Conflicts of Interest

The authors declare that there are no conflicts of interest relevant to this work.
